# Primiparous women’s experiences of midwife-led continuous labor support in Ethiopia: a qualitative study

**DOI:** 10.1080/16549716.2026.2726658

**Published:** 2026-09-04

**Authors:** Mengstu Melkamu Asaye, Getie Mihret Aragaw, Kihinetu Gelaye Wudineh, Eden Bishaw Taye, Kerstin Erlandsson

**Affiliations:** a Department of Women and Family Health, School of Midwifery, College of Medicine and Health Sciences, University of Gondar, Gondar, Ethiopia; b Department of general midwifery, School of Midwifery, College of Medicine and Health Sciences, University of Gondar, Gondar, Ethiopia; c Department of Midwifery, College of Medicine and Health Sciences, Bahir Dar University, Ethiopia; d Department of Clinical Midwifery, School of Midwifery, College of Medicine and Health Sciences, University of Gondar, Gondar, Ethiopia; e School of Health and Welfare, Dalarna University, Sweden

**Keywords:** Midwife-led support, midwifery model of care, continuity of care, childbirth experience, Ethiopia

## Abstract

**Background:**

Midwife-led continuous labour support is a woman-centered approach that has been shown to improve childbirth outcomes. Despite strong global recommendations, it has yet been implemented in Ethiopia.

**Objective:**

This study aimed to explore the experiences of primiparous women with midwife-led continuous labour support in northwest Ethiopia.

**Methods:**

A qualitative exploratory design was used from July to December 2024. In-depth interviews were conducted with 16 primiparous women who received midwife-led continuous labour support in four primary hospitals. Interviews were audio-recorded, transcribed verbatim, translated into English, and analyzed using latent content analysis.

**Results:**

Four sub-categories emerged: emotional empowerment and mindset transformation; compassionate, respectful, and welcoming care; physical and informational support enabling coping and comfort; and a desire for expanded support and system strengthening. Participants considered this model superior to routine care for achieving a positive birth experience, and they advocated its wider adoption, family involvement, and investment in midwives.

**Conclusion:**

Midwife-led continuous labour support fostered positive birth experiences and strengthened relationships between women and midwives. As the mothers reported, the support enhanced their confidence, emotional empowerment, and development of strategies to cope with labor pain. This approach has clear clinical and policy implications for improving maternal and newborn care. This approach is superior to routine care and is strongly recommended by these mothers as standard practice, offering opportunities for family involvement and institutional investment in midwives.

## Paper Context

- **Main findings:** Midwife-led continuous labour support improved the childbirth experiences of primiparous women in four primary hospitals in northwest Ethiopia.
- **Added knowledge:** Continuous labor support by midwives fosters women’s confidence, trust in their providers, emotional empowerment, and effective coping with labour pain, and has the potential to reduce unnecessary obstetric interventions.
- **Global health impact for policy and action:** Implementing midwife-led continuous labour support strengthens the woman–midwife relationship and improves care quality. These findings support scaling up this approach within Ethiopia and in settings with similar maternal-health system contexts.

## Background

Midwife-led continuous labor support is a woman-centered model of care that embodies a philosophy and deliberate approach to supporting women during labor [1]. It is founded on a collaborative relationship between the woman and the midwife, grounded in shared humanity, mutual recognition, and respect for each other’s expertise [2]. According to the World Health Organization (WHO), positive birth outcomes require effective interaction between women and midwives, characterized by respect, clear communication, and emotional support throughout pregnancy and childbirth [3]. Midwife-led continuous labor support includes physical, psychological, mental, and emotional care tailored to women’s wishes, enhancing mutual trust and fostering positive experiences [4]. Women who receive such support report a reduced need for pain relief and greater satisfaction with childbirth [5]. And the continuous presence of midwives, combined with empathy and understanding, improves women’s mental well-being during labor [6]. Prioritizing midwifery care that respects the physiology of childbirth, minimizes unnecessary interventions, and ensures ongoing physical and emotional support has been shown to increase spontaneous vaginal birth rates [7]. Midwife-led continuity of care has been shown to increase vaginal delivery rates and reduce cesarean section rates [8–11], increasing vaginal delivery rates by 25.4% [12].

Ensuring a safe environment, fostering emotional bonds, and demonstrating reliability enable women to develop self-entrustment, gradually building confidence and trust in their midwives [13]. A positive relationship between midwives and women, characterized by kindness and empathy, helps women prepare physically and mentally for childbirth [14]. Continuity of care by midwives, together with appropriate involvement of family members, further enhances positive childbirth experiences, and family support can be effectively guided by midwives to assist women in labor [15]. Despite clear benefits, midwife-led continuous labor support is now uncommon in many hospitals worldwide, including Ethiopia [16]. This is one reason the homebirth rate remains high in Ethiopia [17]. Sometimes other professionals, such as nurses, are involved in antenatal and postnatal care in rural health facilities in Ethiopia, which may affect midwife-led continuity of maternity care [18]. Rural women in Ethiopia report negative experiences with maternity services, including poor quality of care, incompetent and unfriendly health providers, and disrespectful care [19].

Intrapartum maternity care in Ethiopia is provided by midwives; however, they do not offer continuous labor support because of the high caseloads in each maternity ward. There are currently not enough midwives in service to provide one-to-one support during labor. Employing additional graduate midwives to implement continuous labor support is cost-effective, culturally appropriate, and improves maternal and newborn health outcomes [12,20]. These midwives are also assisted during periods of case overload. However, they also require support from other midwives to provide continuous labor care. This approach has not yet been implemented in Ethiopia. Therefore, this study aimed to explore the experiences of primiparous women with midwife-led continuous labor support in northwest Ethiopia.

## Methods

### Design

A qualitative study employing a maximum variation sampling approach was conducted, with data collected through in-depth interviews from July to December 2024 among purposively selected primiparous women who received midwife-led continuous labor support. Ethical approval was obtained on 30 September 2023. However, there was a delay in data collection due to preparation for continuous labor support, provision of training, and implementation of the intervention.

### Setting

The study took place at Debark General Hospital, Dabat Primary Hospital, Dembia Primary Hospital, and Addis-Zemen Primary Hospital. Each maternity ward includes triage, follow-up, second-stage, and postnatal units. The hospitals have small rooms that are not well structured for laboring women according to the stage of labor. Between sixteen and twenty-two midwives work in these intervention wards, alongside physicians and emergency surgeons. Five midwives were assigned at a time. For this intervention, two additional unemployed graduate midwives were recruited in each intervention hospital to provide continuous labor support in case of overload and to ensure adherence to the principle of staying with one woman throughout all stages of labour. According to hospital data, the average monthly birth rate ranges from 115 to 260.

### Intervention

All midwives working in the selected intervention hospitals, together with two additional unemployed graduate midwives, received training in theoretical knowledge, role-play demonstrations, and clinical practice in continuous labor support care. Senior midwives provided training in midwife-led continuous labor support care to all midwives working in the two intervention hospitals, as well as to two unemployed midwives at the intervention sites, who received 2 days of training. The training included theoretical sessions, role-plays, video presentations, and a half-day of clinical practice demonstrations [12]. The trained midwives provided physical support (such as comforting touch, massage, dynamic birth positions, and assistance), emotional support (continuous presence, reassurance, and praise), explanations of procedures, and assistance with informed decision-making. The midwives also helped the woman express her wishes to others and offered pain relief coping strategies. Support included breathing exercises with intervals of more than 20 minutes, 20–40 minutes of continuous back massage over the lower limbs and lower back, physical movement, and the option of various birthing positions. The duration of breathing exercises, massage, and trying different birth positions varied. The intervention was provided frequently, with the total time required exceeding 1–2 hours for women in labor.

### Sampling and participants

Inclusion criteria for midwife-led continuous labor support were primigravida women at 37–42 weeks of gestation, with singleton pregnancies, cephalic presentation, and in the first stage of labor. Primigravida women with obstetric complications such as preeclampsia, hemorrhage, fetal death, or fetal distress at admission, as well as those with documented mental health disorders, were excluded. Thus, primiparous women who met the criteria for the midwife-led continuous labor support intervention and provided written informed consent were included. Primiparous mothers who met the inclusion criteria were approached and invited to participate through face-to-face interviews.

A total of 16 primiparous women living in urban and rural areas were included in the study. Participants’ ages ranged from 18 to 27 years. Most were Orthodox Christians, and two participants were Muslim. Educational levels varied; seven participants had completed primary education, and three held a Bachelor’s degree in education. In the Ethiopian education system, a diploma refers to a three-year college education, and a Bachelor’s degree is a four-year university education.

All participants were married. Occupations included merchants (*n* = 5), government employees (*n* = 5), and one student, with the remaining participants engaged in household or informal work. All participants received continuous labor support from midwives (Table 1).

**Table 1.** Socio-demographic characteristics of the study participants.

Serial No.	Residence	Religion	Education status	Marital status	Occupation
SP01	Urban	Orthodox	Primary school	Married	Merchant
Sp02	Rural	Orthodox	Primary school	Married	Housewife
SP03	Rural	Orthodox	Diploma	Married	Government employed
Sp04	Urban	Orthodox	Secondary school	Married	Merchant
SP05	Rural	Orthodox	Primary School	Married	Farmer
Sp06	Rural	Orthodox	Degree	Married	Government employed
Sp07	Urban	Orthodox	Primary school	Married	Merchant
Sp08	Rural	Muslim	Primary school	Married	Merchant
Sp09	Rural	Muslim	Primary school	Married	Merchant
Sp10	Rural	Orthodox	Diploma	Married	Government employed
Sp11	Urban	Orthodox	Primary school	Married	Merchant
Sp12	Urban	Orthodox	Secondary school	Married	Housewife
Sp13	Urban	Orthodox	Secondary school	Married	Merchant
Sp14	Urban	Orthodox	Degree	Married	Government employed
Sp15	Urban	Orthodox	Diploma	Married	Student
Sp16	Urban	Orthodox	Degree	Married	Government employed

SP = Study participant.

### Data collection

Data were collected through in-depth, face-to-face interviews with primiparous women after childbirth and before discharge. The purpose of the interviews was to explore participants’ experiences and perceptions of midwife-led continuous labor and birth support. The interview guide was developed based on a review of the literature concerning primiparous women’s experiences of midwife-led continuous support in hospital settings. The interviewers and note takers were GMA and EBT, midwifery researchers with experience in qualitative data collection and members of the research team. Participants were asked open-ended questions such as: How do you perceive the service of midwife-led continuous labor and birth support? Can you describe your experience in more detail? Was your support person welcomed at the hospital? If so, how did the staff facilitate this? If not, what barriers did you encounter? Was the continuous presence of your support person helpful during labour? If so, in what way? Were there any negative aspects of having continuous support, and what did they mean to you? Interviews were audio-recorded with participants’ consent. Each interview lasted approximately 60 minutes, allowing sufficient time for participants to describe their experiences in full. The interviews were conducted in December 2024.

### Data analysis

The collected data were analyzed using the content analysis approach described by Elo and Kyngäs (2008) [21], which involves a systematic process of organizing and interpreting textual data to identify patterns and themes. MMA and KE conducted the analysis, which followed three main phases: preparation, organizing, and reporting. During the preparation phase, all audio-recorded interviews were transcribed verbatim. The transcripts were read multiple times to achieve immersion and gain a comprehensive understanding of the participants’ experiences. In the organizing phase, an inductive approach was applied. Meaningful units related to participants’ experiences and perceptions of midwife-led continuous labor support were identified, condensed while retaining their content, and coded. Codes with similar content were grouped into subcategories, which were then further abstracted into a main category. Throughout this process, the researchers constantly compared data within and across interviews to ensure consistency and maintain the authenticity of participants’ accounts. The reporting phase involved describing the categories and illustrating them with representative quotations from the participants to enhance transparency and credibility (Table 2).

**Table 2.** Example of the analysis with meaning units from the original text, condensed meaning units, codes, sub-, and main categories.

Meaning unit from the original text	Condensed meaning unit	Code	Subcategory	Main category
… I was a referral client and was planning to have an operation. However, my support person provided me with continuous emotional support and counselled me to give birth vaginally. I am grateful for their support, as they changed my mindset.	Continuous support and counselling change my mindset.	Emotional support	compassionate, respectful, and welcoming care	A positive childbirth experience
… The service was excellent and exceeded my expectations. The support was very kind, compassionate, and helpful.	compassionate and respectful support	Compassionate support		
… I felt a sense of welcome in this hospital. The midwives are very patient and have a respectful, welcoming approach. I have visited other hospitals, but none like this. Others should practice this kind of support.	Respectful welcoming approach			
The support midwives are very welcoming. They accept me with a friendly and kind approach.	Friendly and kind approach	Friendly support		
The acceptance was respectful and compassionate, and the midwife had a very welcoming approach. This is uncommon in most other hospitals. They deserve thanks.	compassionate and respectful support	compassionate support		
The support midwife encourage and empower me to be free and feel comfort. The welcoming was very good and a friendly approach.	Friendly welcoming approach	Friendly approach		
… guided me to have adequate oral fluids during my laboring time which gave me to gain an energy.	Proving oral fluid to gain energy	Providing oral flued	Physical and Informational Support that Facilitates Coping and Comfort	
… They advised me to drink plenty of fluids during labor and helped me conserve my energy.	Proving oral fluid to gain energy			
They encourage me to have adequate oral fluids that I did consider as it is forbidden before the provision of this midwives led continuous labor support care.	Consideration of fluid intake forbidden previously	Providing oral flued		
… additionally, they did the procedure of back massage and breathing techniques which helped me to decrease labor pain, shorten, and smooth my labor process.	Back massage and breathing techniques for a smooth labor outcome	Non pharmacological relief		
I received continuous labor support care, including back massage and breathing techniques, which helped to reduce my labor pain.	Back massage and breathing techniques decrease labor pain			
She guided me on the position I needed to assume during labor and how I should breathe through it.	the midwife guiding the labor position and breathing	Guiding	Emotional Empowerment and Mindset Transformation	
… It is very valuable and needs to be provided by all healthcare providers. It would also be good if my family could be a support person.	Freedom to select the support families	Needs my family I trust		
I prefer continuous labor support to be provided by individuals with specialized training rather than by midwives, as midwives may become tired and exhausted from also managing the medical aspects.	Needs a support person with special training	Tired and exhausted midwives	Desire for expanded support and system strengthening	
I recommend that if all laboring women are able to receive this type of supportive care, our families could be allowed to be involved in the care.	Need for family involvement to provide support to all women	Family involvement		
The supportive care was very good. This should be part of routine medical care. It could be extended to the pregnancy and postnatal periods.	Continuous labor support could be part of medical care.	Part of routine care		
These midwives deserve recognition and support for their invaluable care. This could enhance the sustainable provision of care.	Needs midwives to be recognition	Recognition of midwives		
It would be nice if my family and friends could be with me in the labour ward, as they might support me alongside the midwives. This could have a positive impact on my birth outcomes.	involvement to provide support to all women	Family involvement		

## Results

Analysis of interview data revealed that women who received midwife-led continuous labor support reported overwhelmingly positive childbirth experiences. During the analysis process, four subcategories were identified: emotional empowerment and mindset transformation; compassionate, respectful care; physical and informational support enhancing coping and outcomes; and a desire for expanded support and system strengthening. The overarching main category, ‘A positive childbirth experience’ emphasized sustained support, including trusted family involvement and recognition of midwives.

## Emotional empowerment and mindset transformation

Emotional support during labor was a critical factor in transforming participants’ attitudes, enhancing self-efficacy, reducing fear, and fostering empowerment, which positively influenced both psychological and physiological experiences.

Continuous emotional support from midwives and companions increased confidence, a sense of safety, and the ability to cope with the challenges of labor. Women emphasized that the presence, encouragement, and compassionate guidance of midwives alleviated fear and reshaped their expectations, shifting from anticipating medicalized births such as cesarean sections to feeling capable of giving birth vaginally. This support shifted mindsets, strengthening internal beliefs in personal birthing capacity.

One woman shared:

My support person provided me with continuous emotional support and counselled me to give birth vaginally … they changed my mindset.

Beyond influencing expectations about the mode of birth, participants highlighted that emotional support fostered a stronger sense of control over their bodies and birth experiences. They reported feeling more resilient in the face of labor pain, less anxious about potential complications, and more confident in their ability to make informed decisions during childbirth. Women also described increased trust-not only in themselves but in the care team –which facilitated open communication and a greater willingness to participate actively in their labor. This transformation extended beyond pain coping strategies, reshaping participants’ perspectives on childbirth. Continuous emotional support encouraged positive self-talk, reinforced personal strength, and built confidence in navigating labor. For many, it led to lasting empowerment and maternal confidence.

## Compassionate, respectful, and welcoming care

Compassionate, respectful, and welcoming care was seen as central to creating a positive, supportive, and emotionally safe environment during childbirth. Participants associated this approach with increased trust, reduced fear, and a greater sense of dignity, highlighting its potential as a model for improving maternity care standards.

Women consistently praised midwives for their respectful, kind, and welcoming approach during labor and childbirth. This dignity and compassion made them feel valued as individuals, not merely patients, in sharp contrast to the impersonal, rushed care found in other healthcare facilities. The relational care-attentive listening, clear explanations of procedures, and patient responses to questions – was both reassuring and transformative.

One participant explained:

‘The support was very kind, compassionate, and helpful.’

Another shared:

I felt a sense of welcome … the midwives are patient and respectful. I have visited other hospitals, but none like this.

This relational, respectful care fostered emotional safety, enabling women to relax, focus on labor and express their needs freely. Genuine kindness and empathy reduced anxiety, built trust in midwives, and increased confidence in the birth. Midwives actively acknowledged women’s individuality – their cultural values, preferences, and emotions – creating a holistic impact. Feeling accepted helped women remain calm, tolerate discomfort, and engage positively with support persons, resulting in memorable, affirming experiences that shaped future expectations. Participants urged standardizing this compassionate model across maternity services to enhance emotional well-being, satisfaction, trust, and confidence in facilities.

## Physical and informational support that facilitates coping and comfort

Physical and informational support was described as a cornerstone of continuous midwife-led care, enabling women to manage pain, maintain comfort, and engage actively in the birthing process. By providing practical guidance, encouragement, and reassurance, midwives facilitated a safer, more positive, and empowering childbirth experience, reinforcing the critical value of continuous, individualized care.

Participants valued midwives’ hands-on support for coping and comfort during labor: guidance on optimal positions, breathing techniques, encouragement to stay hydrated, and non-pharmacological relief such as back massage and relaxation. These interventions reduced discomfort, empowered women with a sense of control, and promoted active participation in the birth. Many found these approaches novels, in contrast to the limited practices in other facilities.

One participant explained:

They guided me on the position I needed to assume and how to breathe.

Another shared:

“Back massage and breathing techniques helped reduce my pain and made my labor process ’smoother’.” Women reported that these physical and informational interventions contributed to greater comfort, sustained energy, and smoother labour progression. They described feeling reassured that the midwives were attentive to their physical and emotional needs, providing guidance tailored to each stage of labour. Participants highlighted that receiving continuous, skilled support in this way made them feel prepared and confident, as they understood what to expect, how to manage contractions, and how to conserve energy for active pushing.

In addition, the combination of practical support and information was seen as mutually reinforcing. Learning and applying coping techniques while receiving ongoing encouragement enhanced women’s self-efficacy and reduced anxiety, creating a positive feedback loop between physical comfort and emotional resilience. Participants emphasized that this integrated approach – blending hands-on interventions with clear, timely guidance – was instrumental in helping them feel supported, capable, and in control throughout labour.

## Desire for expanded support and system strengthening

Participants urged the integration of continuous labor support into routine maternity care, extending it antenatal and postnatal to improve preparedness, confidence, and resilience, resulting in safer, more positive experiences. They advocated involving trusted family members alongside providers to offer emotional comfort and reassurance.

One participant shared:

It would be good if my family could be a support person … This could have a positive impact.

Women recognized the challenges midwives face in high-volume settings, such as fatigue from heavy caseloads and multiple duties. They suggested that trained family members could assist under midwife guidance, easing workloads while ensuring continuous, quality care.

A participant explained:

Continuous labour support could be provided by family members who have received specific training from midwives. Because the midwives are work loaded.

In addition to practical solutions for sustaining support, participants emphasized the importance of institutional recognition and formal support for midwives. They noted that acknowledging midwives’ efforts, ensuring adequate staffing, and providing resources could help maintain high-quality care, prevent burnout, and ensure that the benefits of continuous support are preserved for all women. One woman remarked:

‘These midwives deserve recognition and support for their invaluable care.’

Participants emphasized that continuous labour support should not be an optional or ad hoc service but an integral part of maternity care. They advocated scaling up the model, providing structured training for healthcare providers and family members and institutionalizing policies to support midwives in delivering this essential care. These recommendations reflect women’s recognition of the value of continuous support and the systemic requirements needed to sustain and expand it effectively.

## Discussion

This study suggests that women who received midwife-led continuous labor support experienced profound emotional, physical, and relational benefits. This support increased confidence, reduced pain, and enhanced the overall childbirth experience. Participants considered this care as superior to routine services and strongly advocated for it to become standard practice, with opportunities for family involvement and institutional investment in midwives.

The midwife-led continuous labor support intervention includes physical support (such as comforting touch, massage, dynamic birth positions, and assistance), emotional support (continuous presence, reassurance, and praise), explanation of procedures, and help with making informed decisions. This is consistent with previous studies showing that midwives also assisted women in expressing their wishes to others and provided pain relief and coping strategies, all of which contributed to improved labor and birth outcomes [9,20,22]. This explains that if the support improves birth outcomes, and then, the mother develops a positive birth experience. Primiparous mothers described that continuous emotional support from midwives helped them feel confident, safe, and able to cope with labour. It empowered women to participate in shared decision-making and develop coping strategies for labour pain. Midwife-led continuous support fostered confidence, safety, and psychological readiness, enabling the women to cope better with labour. Similarly, other studies have shown that midwife-led support reduces pain and increases satisfaction with childbirth experiences [9,20]. Furthermore, the midwives’ continuous presence, empathy, and emotional understanding contributed to improved mental well-being [6] and strengthened trust [23]. Primiparous women in particular reported increased confidence, supported by findings that midwives’ empathy and spiritual care promote positive childbirth experiences and confident mothering [24]. This enabled mothers to have positive childbirth experiences. This suggests that midwife-led continuous labor and childbirth support is vital and should be implemented and strongly encouraged in all maternity care facilities. It helps to improve maternal and newborn outcomes and could serve as a useful indicator for scaling up the intervention. There are global calls to expand midwifery care to improve maternal and neonatal outcomes [25]. This indicates that midwife-led continuous labor support is key to reducing unnecessary obstetric interventions [12]. This is also highly relevant for health economics and resources, particularly in Ethiopia as a resource-constrained country. This approach offers value for money by reducing unnecessary interventions and is likely to improve maternal and newborn health outcomes, which can help prevent costly adverse events and prolonged hospital stays.

Continuous emotional and relational support also transformed expectations, with some women shifting from anticipating a medicalized birth to feeling capable of a vaginal birth – an outcome supported by the literature linking midwife-led care with increased rates of spontaneous vaginal birth and reduced unnecessary interventions [7]. This can reduce maternal and newborn procedural complications related to instrumental-assisted delivery, as well as the length of hospital stay. Effective communication further enabled maternal self-trust and emotional safety [13]. It allowed the mother to develop trust and confidence in midwives and helped her to build self-confidence and emotional control strategies.

Concerns about the dehumanization of childbirth have led to renewed calls for continuous midwife-led support during labour [26]. Integrating midwife-led continuous support into routine care that is framework-based, culturally appropriate, and context-specific could address dehumanization and related challenges, and improve maternal experiences. The midwives compassionate and respectful care gave the women a positive childbirth experience. Women valued the midwives’ kindness and respectful approach, describing this care as deeply woman-centered. This aligns with the philosophy that midwife-led support is built on mutual respect and shared humanity [2] and with WHO recommendations promoting respectful communication and emotional support for positive birth outcomes [3]. This suggests that midwife-led continuous labor support is woman-centered and positively influences the development of positive childbirth experiences. Thus, it is time to implement the intervention in all maternity wards in Ethiopia and other similar settings.

Women felt seen, valued, and treated with dignity –experiences supported by international recommendations that prioritize midwife-led continuous support [27]. This approach enables high-quality care and supports evidence that positive midwife–woman relationships enhance both physical and emotional preparation for birth [14]. It helps primigravida women become more stable, prepared, confident, and experience positive childbirth. These findings may have clinical and policy implications for scaling up such support for all laboring women in all health facilities.

Informational support helped women prepare mentally and physically for childbirth and early motherhood [13]. Family involvement, when guided by midwives, also enhanced women’s comfort and supported positive childbirth experiences, consistent with the literature showing that choosing companions increases satisfaction and cultural connectedness [15]. This suggests that support can also be provided by family members, with the guidance or training from midwives for those who will attend the laboring women. Women emphasized the value of practical, hands-on support, including labour positions, breathing guidance, encouragement to hydrate, and non-pharmacological pain relief such as back massage. This reflects evidence that midwives are central providers of labour pain education and individualized coping strategies [16]. It is recommended that all women in all health facilities receive the intervention, as demonstrated by the primiparous mothers who received continuous labor support from midwives in this study.

Women described midwife-led continuous labor support interventions as empowering and sometimes novel, in contrast to their previous experiences of restricted care. Such support enhances coping by providing a safe environment and reducing fear and vulnerability [28], while midwives’ expertise enables tailored pain-coping techniques [29]. Primiparous women advocated for expanded support and strengthened health facility systems. Midwife-led continuous support offers a comprehensive, woman-centered approach that promotes confidence, empowerment, and positive childbirth experiences, with implications for improving quality of care and reducing maternal and newborn morbidity and mortality, as midwives have the potential to prevent millions of maternal and neonatal deaths worldwide [16]. This is highly significant, given the global calls to scale up the provision of midwifery care [25].

Women advocated for continuous support across antenatal, intrapartum, and postpartum periods. This aligns with global calls to expand midwifery care and evidence demonstrates the feasibility of implementing and sustaining continuous midwife-led support in clinical settings [30]. Family involvement remained a key preference, reinforcing findings that choosing companions can enhance coping and emotional belonging [9]. Midwives’ continuous labor support encourages primiparous mothers to cope with labor pain, and it is recommended for implementation for all laboring mothers in Ethiopia.

## Strengths and limitations

To ensure trustworthiness [31], two researchers, MMA and ER, members of the research team, conducted the analysis independently, followed by discussion to reach consensus. This systematic approach enabled a rigorous exploration of primiparous women’s experiences and perceptions of midwife-led continuous labour and birth support in the selected hospitals. Credibility was ensured through collaborative coding, analysis, and interpretation by Authors 1–5. Transferability was supported by providing rich, detailed descriptions and sufficient contextual information, participant characteristics, and a clear description of the research process, enabling readers to assess the applicability of the findings to other settings. Dependability and confirmability were strengthened by illustrating findings with direct quotes and involving multiple authors in the analysis to ensure that the themes reflected the data. Reflexivity was maintained, as the interview guide and all stages of analysis were agreed upon by at least two authors to enhance objectivity. Although the study was small scale, we achieved depth in the interviews and reasonable diversity among participants who had experienced this service and had different sociodemographic characteristics. The two interviewers and notetakers were GMA and EBT, midwifery researchers with experience in qualitative data collection and members of the research team.

## Conclusion

Midwife-led continuous labour support fostered positive birth experiences and strengthened relationships between women and midwives. As the mothers mentioned, the support enhanced their confidence, emotional empowerment, and the development of strategies for coping with labor pain. This approach has clear clinical and policy implications for improving maternal and newborn care. Participants experienced and described this approach as superior to routine care and strongly recommended it as standard practice, alongside opportunities for family involvement and institutional investment in midwives.

## Clinical implications

Findings on midwife-led continuous labor support should inform policy, practice, and future research. Further research is needed to explore how midwife-led continuous labour support could be scaled up in Ethiopia, considering human and economic resources, existing professional norms, and the recognition of midwives. Participants also suggested that midwife-led care should be expanded into antenatal and postnatal care services.

## Supplementary Material

COREQ checklist.docx

## Data Availability

This manuscript contains all data generated or analyzed during the study. As this is a qualitative study, all data are included in the manuscript.
